# Production of Anthocyanin-Enriched Juices by Electrodialysis with Filtration Membrane Process: The Influence of Duration on Juice Composition, Process Efficiency, and Membrane Fouling

**DOI:** 10.3390/foods13213478

**Published:** 2024-10-30

**Authors:** Eva Revellat, Laurent Bazinet

**Affiliations:** 1 Institute of Nutrition and Functional Foods (INAF), Department of Food Sciences, Université Laval, Quebec, QC G1V OA6, Canada; eva.revellat.1@ulaval.ca; 2 Laboratoire de Transformation Alimentaire et Procédés ElectroMembranaires (LTAPEM, Laboratory of Food Processing and Electro Membrane Processes), Université Laval, Quebec, QC G1V OA6, Canada

**Keywords:** cranberry, polyphenol, electrodialysis, filtration membrane, anthocyanin, fouling, duration

## Abstract

The Electrodialysis with Filtration Membrane (EDFM) system has shown promise in juice enrichment, but further optimization is needed. This study evaluated the effect of processing duration (3 and 6 h) on juice composition, process efficiency, and membrane fouling. Results demonstrated a significant impact of processing time on juice composition, especially anthocyanin and mineral content. Two anthocyanin-depleted juices (−18.94% and −30.70%) and two anthocyanin-enriched juices (26.21% and 44.21%) were produced. Similar energy (1512.13 Wh/g of anthocyanins) was required to migrate equivalent amounts of anthocyanins over both time periods, with no impediment due to fouling observed, although the system’s resistance increased (2.5-fold after 3 h, 3.2-fold after 6 h). Membrane fouling was characterized through conductivity, thickness, ATR-FTIR, SEM-EDX, and foulant identification. Minimal anthocyanin accumulation occurred on cation-exchange membranes (CEM), while anthocyanins and PACs concentrated within the filtering layer of filtration membranes (FM). However, fouling did not increase with longer processing. Structural alterations were noted in anion-exchange membranes (AEMs), suggesting instability under high electric fields. Overall, EDFM effectively enriched cranberry juice with anthocyanins, but further research is necessary to address AEM degradation under limiting current density conditions.

## 1. Introduction

Anthocyanins are a sub-group of the polyphenol family and are formed of an aglycone and a glycoside [1]. Numerous health benefits are attributed and reported in the literature concerning anthocyanins, e.g., anti-cancer effects [2,3], cardiovascular prevention [4,5], the reduced risk of urinary tract infection [5], and also a recently discovered protective effect against neurodegenerative diseases through the microbial–intestinal–brain axis [6]. Anthocyanins can be found in cranberry [7], which is mainly consumed as juice [8].

Bazinet et al. [9] used an electrodialysis with filtration membrane (EDFM) system to obtain juices with different anthocyanin concentrations. This process has shown promising enrichment results [9], and in a recent study different filtration membranes were tested in the EDFM system for the enrichment of cranberry juice [10]. The best membrane in terms of anthocyanin enrichment was selected by pinpointing the membrane properties that influenced the migration rate of anthocyanins [10]. To delve deeper and enhance the process further, it is necessary to evaluate the influence of duration on the process to ascertain its effects on juice composition, process efficiency, and membrane fouling. Indeed, EDFM is a new green process, and the fouling mechanism in this type of system is less detailed than classical electro-membrane processes. In addition, membrane fouling and process efficiency remain critical challenges in the optimization of juice enrichment processes.

Membrane fouling is defined as the adsorption or deposition of undesirable molecules on the surface of a membrane or inside the membrane due to different interactions (colloidal, mineral, biological) [11]. Fouling increases the electrical resistance which decreases the permselectivity and alters the membrane integrity, which results in higher energy consumption and lifetime limitation of the process [11,12]. Numerous studies have been conducted on the fouling of the ion exchange membrane (IEM) because it is a major problem in ED [11,13,14,15]. Thus, extensive studies of the fouling of the IEM by polyphenols identified these main interactions: hydrophobic interaction (π-π stacking between aromatic ring), hydrogen bonds, and electrostatic interactions between the foulant and fixed sites of the membrane and the membrane polymer itself. The authors also highlighted the penetration of polyphenols inside the IEM and the formation of colloidal particles, even in relatively small membrane pores [14], but also the formation of island-like foulant layers [16]. Furthermore, extended duration of process can worsen fouling and/or might affect fouling differently from previous studies, since polyphenols may adsorb onto membrane surfaces and penetrate the matrix through these mechanisms [11,14,16]. However, the fouling of FM has mostly been described for pressure-driven processes [17,18] and focused on the impact of membrane chemical composition and other membrane physicochemical properties such as pore size. Similar interactions for IEM are described in the literature between polyphenols and FM in pressure-driven processes [19]. In pressure-driven processes, prolonged filtration decreases membrane flow rates, necessitating cleaning procedures to restore performance [20]. Thus, duration plays a crucial role in fouling severity and reversibility, with longer processing times often leading to more compacted fouling layers and stronger foulant–membrane interactions. And in the EDFM process where no pressure is applied, the evolution of the FM fouling by polyphenols over time has never been assessed. In this context, the objectives of the present work were (1) to study the evolution of the juice physicochemical parameters during the EDFM process, (2) to evaluate the performances of the EDFM process, (3) to characterize membranes before and after 3 or 6 h of treatment, (4) to identify the polyphenols that interact with the IEM and FM, and (5) to understand the fouling mechanism and how it impacts the process.

## 2. Materials and Methods

### 2.1. Materials

#### 2.1.1. Membranes

Neosepta homogeneous membranes CEM and AEM were both purchased from Ameridia (Napa, CA, USA) and used in the system, as well as a PVDF 250 kDa (Synder, Vacaville, CA, USA). The selection of the PVDF 250 kDa membrane was based on the recent results of Revellat et al. [10], who demonstrated that this membrane amongst 5 other commercially available membranes had the best performance in terms of anthocyanin migration. Information regarding the chemical structures of AEM and CEM have lately been reported and detailed by Perreault et al., Chen et al., and Luo et al. [13,21,22]. The supplier provided detailed information on the chemical structure of the FM, indicating that the filtering layer is made of PVDF, and the support layer consists of non-woven polyester.

#### 2.1.2. Cranberry Juice

A 15 kg bucket of 8° brix cranberry juice provided by Fruit d’Or (Plessisville, QC, Canada) was thawed, then aliquoted in bottles of 2 L and stored at −20 °C. Two days before the EDFM treatment, one bottle was thawed and stored at 4 °C. 

### 2.2. EDFM Configuration

**Figure 1:**
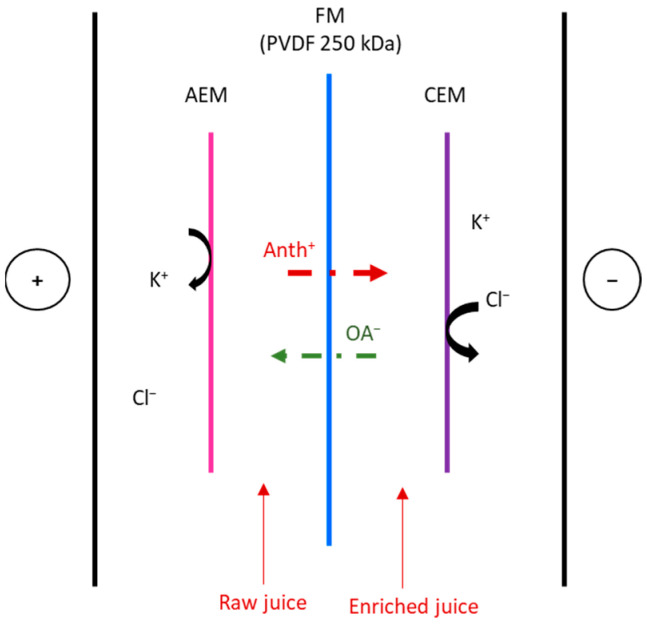
Schematic representation of the EDFM configuration used for the juice enrichment.

The EDFM configuration (Figure 1) consisted of one CEM, one AEM, and one PVDF 250 kDa membrane in a 10 cm^2^ effective electrode surface microflow cell (ElectroCell AB, Karlskoga, Sweden). A 316 stainless steel electrode was used for the cathode and a dimensionally stable electrode (DSA) for the anode. An electric power supply (BK Precision 9110, Yorba Linda, CA, USA) furnished the anode/cathode voltage difference. To allow the recirculation of the solutions, the system was composed of 3 closed loops. The flow rate of the solutions was maintained thanks to three centrifugal pumps (Iwaki Magnet Pump, Iwaki Co., Ltd., Tokyo, Japan) and three flow meters (Aaakborg Instruments and Controls, Inc., Orangeburd, SD, USA).

To evaluate the impact of the process duration, 300 mL of juice was circulated in the two central compartments with a flow rate of 100 mL/min for 180 min or 360 min. These durations were selected based on findings from a previous study, which showed that after 180 min. of EDFM (using the same FM), anthocyanin enrichment reached 21.5% [10]. Shorter durations were insufficient for achieving a significant interesting enrichment. To assess whether extending the treatment time would further enhance enrichment without negatively affecting the process and to understand how the extended duration may impact the overall efficiency, it was decided to double this treatment duration.

Thus, in one compartment, the juice anthocyanin concentration will decrease while the other one will increase. In the electrode rinsing compartment, a 20 g/L KCl solution was circulated at a flow rate of 400 mL/min, as previously done by [9]. A constant voltage difference of 30 V was chosen to replicate the conditions, demonstrating high juice polyphenols enrichment following EDFM, set by Bazinet et al. [9] for comparing results. The distance between the two electrodes was 2 cm. All along the EDFM, the conductivity, pH, intensity, and voltage were measured. Three replicates of each EDFM were done. Samples of each juice were aliquoted each hour to follow the anthocyanin, proanthocyanidin, mineral, and organic acid concentrations. 

### 2.3. Analyses

#### 2.3.1. Juice Physicochemical Parameter Evolution During EDFM

*pH and conductivity*

The pH of both raw and enriched juices was measured during EDFM, using a pH-meter (Models Orion Star A221, Thermo Scientific, Waltham, MA, USA).

The conductivity of the juices was measured with a YSI conductivity meter (Model 3100) using a YSI immersion probe (Model 3252, cell constant K = 1 cm^−1^; Yellow Springs Instruments, Yellow Springs, OH, USA). 

*Anthocyanin content and migration rate*

The concentration of anthocyanins in the juices was analyzed by HPLC, as described by Wu and Prior [23]. Samples were first diluted (dilution factor = 1.25) in a 0.5% TFA methanol solution and filtered on a 0.22 μm nylon filter. An elution time of 1 mL/min was applied. The HPLC was supplied with a ZORBAX SB-C18 column (Agilent, 5 µm, 4.6 × 250 mm, Santa Clara, CA, USA) associated with a DAD detector (detection wavelength of 520 nm). The 100% methanol solution and a water/formic acid (95%/5%) were used as mobile phases. The cyanidin-3-glucoside (MW = 449.2 g/mol and ε = 26,900 L/mol·cm) was used as standard. At the end of the EDFM treatment, global and individual anthocyanin migration rates (g/m^2^·h) were calculated by dividing the total quantity of anthocyanins (g) migrated toward the cathode by the effective surface area of FM (m^2^) and the duration of the EDFM process (h) according to Equation (1).

(1) $$Anthocyanin\;Migration\;rate=\frac{\left(C_{f}-C_{0}\right)\times V_{EJ}}{A\times t}$$

$C_{f}$ and $C_{0}$ (g/L) are the final and initial concentrations in anthocyanins of the enriched juice, $V_{EJ}$ (L) is the enriched juice volume, *A* (m^2^) is the area, and *t* (h) is the treatment duration.

*Proanthocyanidin content*

Proanthocyanidin concentrations were analyzed by HPLC, as detailed by Khanal et al. [24]. Samples were diluted (dilution factor of 200/59) in 0.71% acetic acid acetone solution and filtered on a 0.22 μm nylon filter. 

*Organic acid content and migration rate*

The concentration of organic acid (OA) in the juices was analyzed by HPLC, as described by Serre et al. [25]. Samples were first diluted (dilution factor = 5) in DI water and filtered on a 0.22 μm nylon filter. The HPLC system was equipped with a Synergi Hydro-RP 80A column (250 mm x 4.6 mm, Phenomex, Torrance, CA, USA) and a UV detector (detection wavelength of 214 nm). A 0.8 mL/min flow rate was used. The mobile phase was a 0.2 M KH2P04 solution at pH 2.4. A total of 10 μL of samples were injected. To determine calibration curves and retention times, and therefore the organic acid concentrations, malic, citric, and quinic acid standards (Sigma-Aldrich, Saint-Louis, MO, USA) were selected. OA migration rates (g/m^2^·h) were calculated by dividing the total quantity of organic acid (g) migrated toward the anode at the end of the EDFM by the effective surface area of FM (m^2^) and the duration of the EDFM process (h) according to the following equation:

(2) $$Organic\;acid\;migration\;rate=\frac{\left(C_{f}-C_{0}\right)\times {\;V}_{EJ/RJ}}{A\times t}$$

$C_{f}$ and $C_{0}$ are the final and initial concentrations in organic acids of the enriched or raw juice, $V_{EJ}$ and $V_{RJ}$ (L) are the enriched or raw juice volumes, respectively, *A* (m^2^) is the area, and *t* (h) is the treatment duration.

*Mineral content*

The initial and final raw and enriched juice mineral concentrations were determined by ICP-OES (Agilent 5110 SVDV Agilent Technologies, Victoria, Australia) [21]. The wavelengths used were 396.847; 393.366; 422.673; 317.933 nm for calcium, 766.491; 769.897 nm for potassium (K), 257.610; 259.372; 260.568; 294.921 nm for magnesium (Mg), 589.592; 588.995 nm for sodium (Na), 213.618; 214.914 nm for phosphorus (P), and 213,598; 224,700; 324,754; 327.395 for copper (Cu). The analyses of these ions were performed in axial and radial views. The analysis was carried out in triplicate. 

*Juice color*

The color of the juice was analyzed with a Minolta chromameter (Model Minolta meter CR-300, Konica Minolta Inc., Mississauga, ON, Canada). The juice color is described with three parameters L* (lightness), a* (color intensity differing from red to green), and b* (color intensity differing from yellow to blue) [9]. 

*Degree brix*

A digital hand-held refractometer PAL-1 (ATAGO, Tokyo, Japan) was used to determine the degree brix of both raw and enriched juices.

#### 2.3.2. Electrodialytic Parameters—Process Performance

*Resistance*

The global system resistance (R) was calculated with Ohm’s law (U = R·I). The voltage (U) and current intensity (I) were read from the power supply (BK Precision 9110, Yorba Linda, CA, USA).

*Relative energy consumption*

The relative energy consumption (REC) in Wh/g of anthocyanins transported was calculated after 3 and 6 h of treatment according to the following equations:

(3) $$REC=\frac{(U\int_{0}^{t}I\left(t\right)dt)/3600}{\left|C_{t}-C_{0}\right|\times V_{EJ}}$$

$C_{t}$ and $C_{0}$ are the concentrations of anthocyanins in the enriched juice at times t and 0, $V_{EJ}$ is the volume of enriched juice (L), I the current (A), t is the process duration (h), and U the voltage (V). 

#### 2.3.3. Characterization of Membranes 

*Thickness*

The thickness of the PVDF 250 kDa, CEM, and AEM membranes was measured using an electronic digital micrometer (Marathon Watch Company Ltd., Richmond Hill, ON, Canada). The thickness was measured six times at different points of the membrane [25].

*Ionic conductance and conductivity*

CEM and AEM were equilibrated in 0.5 M NaCl solution for 30 min before measurement. The FM conductivity was not measured after treatment to avoid the loss of the foulant once in contact with the salt solution. Indeed, the higher pore size of FM can help the desorption of the foulant in comparison with an IEM. A YSI conductivity meter (Model 35; Yellow Springs Instrument Co., Yellow Springs, OH, USA) connected to a designed clip from the Laboratoire des Matériaux Échangeurs d’Ions (Université Paris XII, Créteil, France) was used to measure at six different positions the ionic conductance (G). Electrical resistance (R) can be obtained thanks to the electrical conductance (G) measurement (G = 1/R). The transversal electrical resistance of the membrane $R_{m}$ Ω) can be obtained using the following equation:

(4) $$R_{m}=R_{m+s}-R_{s}$$

where $R_{s}$ (Ω) is the resistance of the reference solution and $R_{m+s}$(Ω) is the overall resistance. 

Taking into account the thickness of the membrane, the specific electrical conductivity (κ, in S/cm^−1^) can be obtained as follows [26]:

(5) $$\kappa =\frac{l}{R_{m}A}$$

where $R_{m}$ (Ω) is the transversal electrical resistance of the membrane, A (=1 cm^2^) is the measurement, and l (cm) is the thickness of the membrane. 

*Optical microscopy and membrane cross-section visualization*

All membranes were cut with a cryostat microtome (HM 500 OM, Microm International GmbH, Walldorf, Germany) and put in water between a microscope cover glasses and glass slides. Images of the membrane cross-sections were taken in brightfield microscopy at 20× with a Zeiss Axio observer (Carl Zeiss, Toronto, ON, Canada) with a motic camera. 

*FTIR*

FTIR spectra were obtained with a Nicolet 6760 (Nicolet Instrument Corp., Madison, WI, USA) and an attenuated total reflection accessory (ATR). A total of 128 scans were performed, with a resolution of 4 cm^−1^. The wavelength range was from 4000 to 650 cm^−1^ to analyze the same range presented in the literature for organic foulant (polyphenols) [16]. All data processing was performed using the Omnic spectra software v5.1a (Nicolet Instrument Corp., Madison, WI, USA). The baseline was subtracted from the sample spectra and all spectra were normalized. Both sides of the dried CEM, AEM, and FM (pristine and used membranes) were analyzed for each repetition. 

*SEM—EDX*

Prior to SEM analyses, to ensure all water present inside the membrane was removed, 25 mm^2^ coupons were dried at ambient temperature more than a week before analysis. Images of both sides of the CEM, AEM, and FM (pristine and used membranes) were taken using a scanning electron microscope (SEM) (Quanta 3D FEG, ThermoFisher, Hillsboro, OR, USA) [27]. A 5 kV accelerating voltage with a 9.5–10.5 mm working distance 400× magnification was used. The surface elemental composition was determined thanks to an energy dispersive spectrometer (EDX) (PV8206/60 Genesis XM2, EDAX, Japan). Analyses were performed on each membrane and repetition. 

*Extraction of polyphenols*

**Desorption procedure**

After each EDFM, a coupon of 8 cm^2^ of PVDF 250 kDa membrane and 4 cm^2^ of AEM and CEM was cut into small pieces before desorption and weighted. To obtain a solution at 4% (m/V), an appropriate volume of a solvent solution composed of 25% acetonitrile/25% methanol/25% isopropanol/25% water was added to the piece of membrane in a 5 mL Eppendorf. Such an extraction of anthocyanins was previously described by Bdiri et al. [14]. Then, coupons of membranes and solvent solution were agitated for 24 h on a rocker (Fisher) at a speed of 30 and an angle of 15°. After five-minute centrifugation at 6900 rpm, 100 µL of the final volumes of AEM and CEM supernatant were aliquoted in 1.5 mL Eppendorf for total polyphenol analysis. The remaining volumes were aliquoted in equal volumes of supernatant in two 2 mL Eppendorf’s for anthocyanins and PACs analyses. The supernatants were vacuum-dried (Savant SPD131DDA SpeedVac concentrator, Thermo Scientific, Waltham, MA, USA). Analyses were performed on each membrane and each repetition. 

**Total polyphenol content**

Before analysis, dried extracts were dissolved in 50 μL of distilled water. As standard solution, acid gallic was used at concentrations of 0.5; 5.0; 10; 25; 50; 100; 250; and 500 mg/L. A total of 20 μL of distilled water (blank), sample, and standard solution were added to wells of a 96-well microplate. Then, 100 μL of 1:10 diluted Folin-Ciocalteu solution was added to the wells [28]. To stop the reaction between the Folin-Ciocalteu solution and the samples, 80 μL of a 7.5% sodium carbonate solution was added to the wells. An incubation of 60 min at ambient temperature was done before measurement. A xMark Microplate spectrophotometer (BioRad, Mississauga, ON, Canada) was used to measure the absorbance of each well at 765 nm. The total phenolic content was indicated as mg gallic acid equivalent/g of sample. 

Results were presented in μg of polyphenol/cm^3^ of membrane, based on the membrane thickness measurements.

(6) $$\frac{m_{p}}{V_{m}}=\frac{C_{p\;\times \;V_{s}}}{V_{m}}$$

With $m_{p}\;\left(\mu g\right)\;$the mass of polyphenols, $V_{m}$ (cm^3^) the volume of membrane, $C_{p}$ (μg/L) the concentration of polyphenol obtained by spectrophotometry, and $V_{s}$ (L) the volume of solution added to the dried extract.

**Anthocyanin and PAC contents**

The concentrations of anthocyanin and PACs in the desorption solutions were analyzed by HPLC as described in Section 2.3.1. Prior to the HPLC analysis, dried extracts were diluted in 0.5% TFA methanol solution for anthocyanins and in 0.71% acetic acid acetone solution for PACs.

Results were presented in μg of anthocyanins or PACs/cm^3^ of membrane, based on the membrane thickness measurements.

(7) $$\frac{m_{A\;or\;P}}{V_{m}}=\frac{C_{A\;or\;P\;\times \;V_{s}}}{V_{m}}$$

With $m_{A\;or\;P}\;\left(\mu g\right)\;$the mass of anthocyanins or PACs, $V_{m}$ (cm^3^) the volume of membrane, $C_{A\;or\;P}$ (μg/L) the concentration of anthocyanins or PACs obtained by HPLC, and $V_{s}$ (L) the volume of solution added to the dried extract.

### 2.4. Statistical Analyses

#### 2.4.1. ANOVA 

Each EDFM condition was carried out in triplicate. The mean value and standard deviation were calculated from the three repetitions. Data obtained for the enriched and raw juices in terms of °Brix, color, concentration (anthocyanins, PACs, AOs, minerals), membrane thickness, and conductivity were treated with a one-way ANOVA and using a Tukey test; significative differences between results were defined at a probability level of *p* < 0.05.

#### 2.4.2. Student *t*-Test

Data obtained for the same juice in terms of pH and conductivity, and migration rate before and after treatment, were compared using a *t*-test; significative differences between results were defined at a probability level of *p* < 0.05.

## 3. Results

### 3.1. Juice Physicochemical Parameters Evolution During EDFM

Four juices were produced and corresponded to the raw and enriched juice compartments (Figure 1) after 3 or 6 h of treatment. The composition and physicochemical characteristics of these juices compared to the initial juice are presented in Table 1.

#### 3.1.1. Mineral Content

A demineralization of the enriched and raw juice was observed for all the minerals except Ca, Cu, and Mg concentrations, which increased after 3 and 6 h for the enriched juice after treatment whereas K, Na, and P concentrations decreased for both juices (Table 1). 

The potassium concentration decreased significantly in the enriched juice with a decrease of 39.1% after 3 h and of 71.5% after 6 h. For the raw juice, the potassium concentration decreased by 74.2% after 3 h and 88.5% after 6 h of treatment. Potassium reached the highest migration with a migration rate of −50.16 ± 2.00 g/m^2^·h for the raw juice after 3 h of treatment due to its high concentration in cranberry juice and because of its high electrophoretic mobility [29] but decreased to −30.25 ± 0.76 g/m^2^·h after 6 h of treatment. Meanwhile, for the enriched juice, the migration rate of potassium was twice lower (−26.64 ± 2.20 g/m^2^·h) after 3 h and stayed after 6 h at an equivalent migration rate (−24.51 ± 1.19 g/m^2^·h). This behavior is in accordance with the EDFM configuration. Indeed, the potassium is migrating from the raw juice through the FM (not selective to mineral species) to the enriched juice quickly after the beginning of the run. Then, the potassium crossed the CEM toward the cathode. Thus, the first juice depleted in potassium is the raw juice, and then the enriched juice. The enriched juice was continuously supplemented in K^+^ as EDFM progressed but the K^+^ concentration in the raw juice decreased leading to a decrease in its migration rate. 

Since the potassium concentration in the juice exceeded that of calcium by thirteen times and magnesium by twenty times, the migration of the other cations was less pronounced until the potassium concentration decreased [30]. Therefore, for the raw juice, except for Cu (with a negligible initial concentration), all the cation contents decreased significantly after 3 h of treatment and even more after 6 h of treatment. These migrations of positively charged minerals from the raw juice to the enriched one were in accordance with the configuration of the system and the migration of cations through the FM toward the cathode. Results also showed an accumulation of Mg and Ca in the enriched juice compartment indicating the hampering of the migration through the CEM. It could be due to the high current density and the subsequent concentration of polarization: migration of divalent ions through the CEM was probably hindered by the facilitated migration of monovalent ions, as previously mentioned by Beaulieu et al., for the demineralization of acid whey by electrodialysis with nanofiltration membrane [30] but also the formation of complexes between the polyphenols and the Mg^2+^ and Ca^2+^ divalent ions at the FM [31].

In the same way, the significant decrease in P anions in the enriched juice after 3 and 6 h was also expected. P migrated easily from the enriched juice through the non-selective FM in the raw juice. Then, it crossed the AEM toward the anode.

#### 3.1.2. Degree Brix and Color

Degree Brix

The degree Brix of cranberry juice can be defined by its total soluble solids (sugars, minerals). Statistical analysis showed a significant change in °Brix between the initial and the final raw and enriched juices after 6 h of EDFM treatments (Table 1). As previously mentioned, sugar molecules did not migrate in the EDFM process (with the electric field as the driving force) [9], but the mineral content decreased in the juices as described previously (see Section 3.1.1). 

Color 

The color parameters (L*a*b) were not significantly different after 3 or 6 h of treatment (Table 1). However, for the raw juice, a* parameter value was higher than for the initial and enriched juice. This may be due to anthocyanin migration from the raw juice to the enriched juice. Bazinet et al. [9] already mentioned that juices with a high anthocyanin concentration had a darker color that impede the measurement of the a* parameter. 

#### 3.1.3. Anthocyanin, PAC, Organic Acid Migration 

*Global anthocyanin migration*

**Figure 2:**
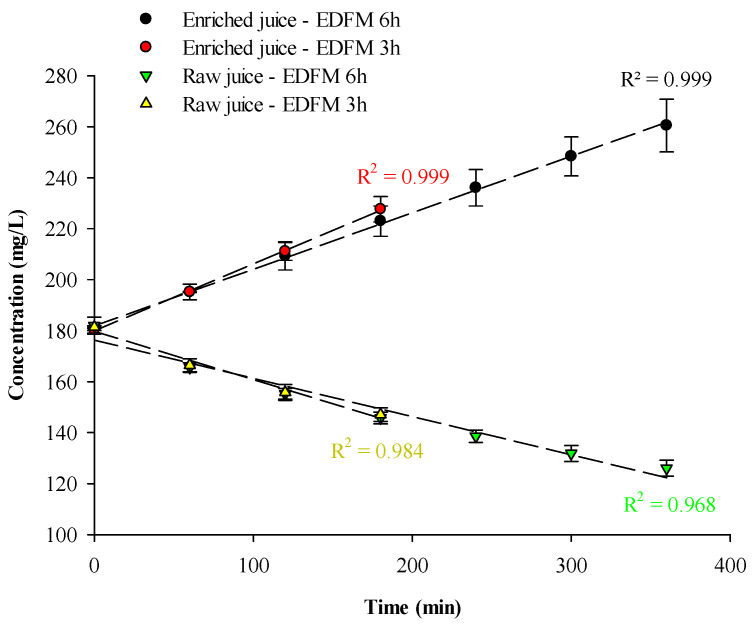
Concentration of anthocyanins as a function of time during EDFM treatment.

A linear migration of anthocyanin was observed (Figure 2), as previously reported by Bazinet et al. [9]. Similar migration rates of 4.72 ± 0.63 g/m^2^·h and 5.31 ± 2.31 g/m^2^·h were achieved for the enriched juice after 180 or 360 min of EDFM, respectively (*p* = 0.692). This migration corresponds to a juice’s enrichment of 26.21 ± 3.70% after 180 min and an enrichment of 44.21 ± 5.36% after 360 min of treatment. Comparable migration rates were also achieved for the raw juice after 180 or 360 min of EDFM (*p* = 1.0. Raw juice’s impoverishment reached −18.94 ± 1.23% after 3 h and −30.70 ± 2.97% after 360 min of treatment. Results suggested that anthocyanin migration was linear and constant all along the treatment, and anthocyanins from the raw juice were all recovered in the enriched juice, suggesting that PVDF 250 kDa did not hinder the migration due to fouling.

*Individual anthocyanin migration*

**Figure 3:**
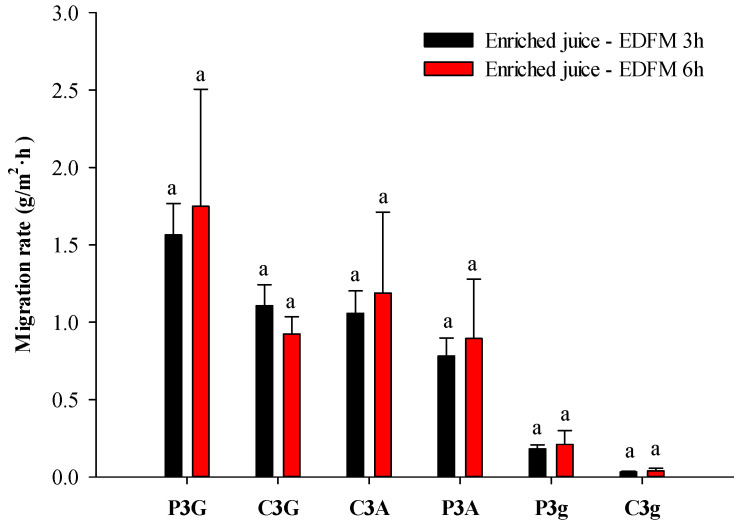
Individual anthocyanin migration rates during EDFM treatment (for the same anthocyanin, values followed with the same letter (a) are not significantly different (*t*-test) at *p* > 0.05).

Individual anthocyanin migration rates are presented in Figure 3. As previously described by Revellat et al. [10], the highest migration rate of individual anthocyanins was correlated to the most abundant individual anthocyanins initially present in the juice. Comparable individual migration rates were achieved for the enriched juice after 3 or 6 h of EDFM for all anthocyanins (*p* > 0.05). 

PAC migrations 

**Figure 4:**
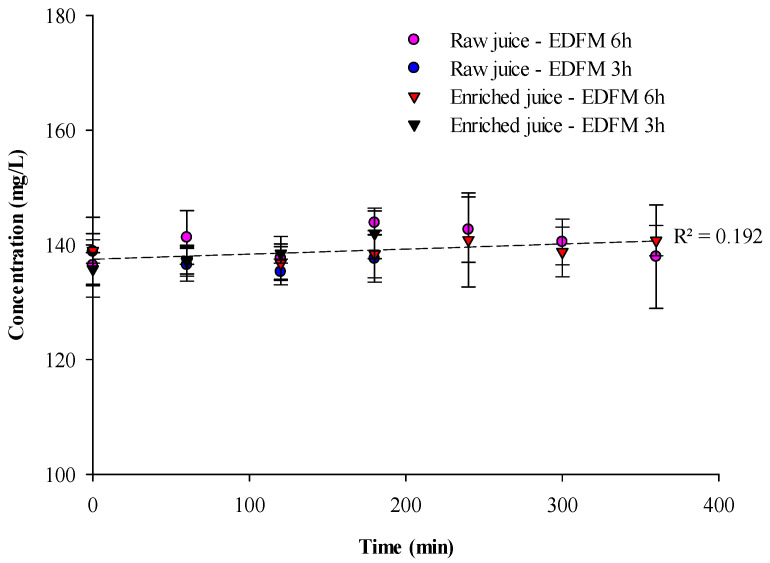
PAC concentration as a function of time during EDFM treatment.

Global PAC concentrations determined by HPLC are presented in Figure 4. For both raw and enriched juice, after 180 or 360 min of treatment, no migration of PACs was observed. These results are in accordance with another study by Revellat et al. [10]. At the pH of the juice, PACs are not charged, thus no migration is expected. 

Organic Acid migrations

**Figure 5:**
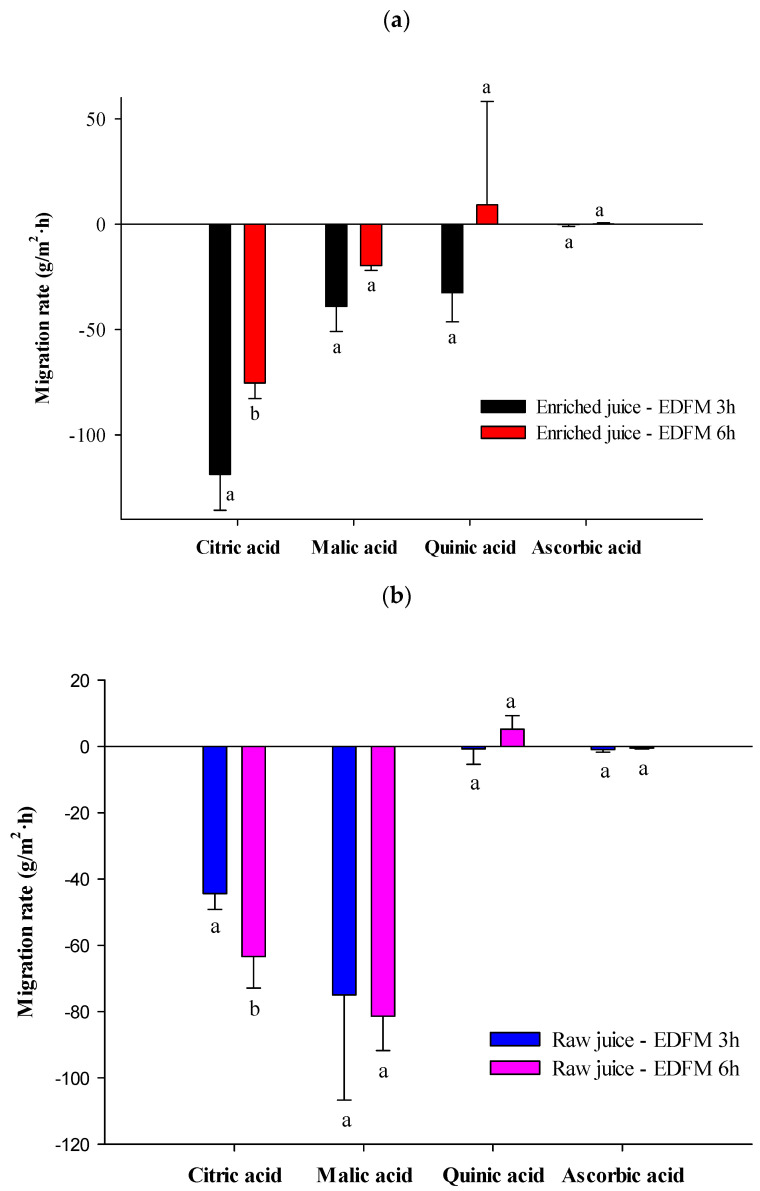
Migration rate of individual organic acids (**a**) for the enriched juice and (**b**) for the raw juice. For the same organic acid, values followed by different letters (a,b) are significantly different (*t*-test) at *p* < 0.05.

Individual organic acid (OA) migration rates are presented in Figure 5. Main organic acids in cranberry juice are quinic acid (molecular weight (MW) = 192.17 g/mol, pKa = 3.46), citric acid (MW = 192.12 g/mol, pKa_1_ = 3.13, pKa_2_ = 4.76, pKa_3_ = 6.39), malic acid (MW = 134.09 g/mol, pKa_1_ = 3.46, pKa_2_ = 5.05) [25]. As already reported in the literature for EDBM [25], a selective migration of organic acid was observed. The enriched and raw juices were depleted in OA (negative migration rate) due to the negatively charged OA which migrated from the juices toward the anode. OA from the enriched juice migrated through the FM, while OA from the raw juice migrated through the AEM. Concerning the enriched juice (Figure 5a), the highest migration rate was reached for the citric acid (−97.07 ± 26.46 g/m^2^·h) (the most abundant organic acid in cranberry juice) due to its lower molecular weight and pKa, then the malic acid (−29.35 ± 13.08 g/m^2^·h). However, the quinic acid migration (the second most abundant organic acid in cranberry juice) seemed to be hindered probably because it has one negative charge, whereas the citric and malic acid have three or two of them, which could explain their higher migration rate. Quinic acid has also an aromatic carboxycilic ring and consequently a higher steric hindrance [25]. Regarding the migration rate of individual organic acids for the raw juice, the highest migration rate was reached for malic acid (−78.15 ± 21.44 g/m^2^·h). Citric acid presented a higher MW = 192.17 g/mol, whereas malic acid has a MW = 134.09 g/mol. Thus, the migration of the citric acid through the AEM was slowed down by steric hindrance of this molecule. 

The evolution of migration through time highlighted that for the enriched juice (Figure 5a) the migration rates were similar after 3 or 6 h of treatment for quinic, malic, and ascorbic acids, but not for citric acid with a migration rate reaching −118.72 ± 17.05 g/m^2^·h after 3 h and a migration rate decreasing to −75.42 ± 7.32 g/m^2^·h after 6 h, and a similar tendency could be observed for the malic acid. In a similar way, for the raw juice (Figure 5b), migration rates for all the organic acids were similar after 3 or 6 h of treatment except for citric acid, but contrary to the enriched juice, the citric acid migration rate increased after 6 h, reaching −63.65 ± 9.53 g/m^2^·h instead of −44.39 ± 4.71 g/m^2^·h after 3 h. After 6 h of treatment, the citric acid concentration in the enriched juice decreased, which could explain the migration rate decline. Similarly, citric acid concentration increased after 6 h in the raw juice and could explain the migration rate increase.

#### 3.1.4. pH and Conductivity

pH

**Figure 6:**
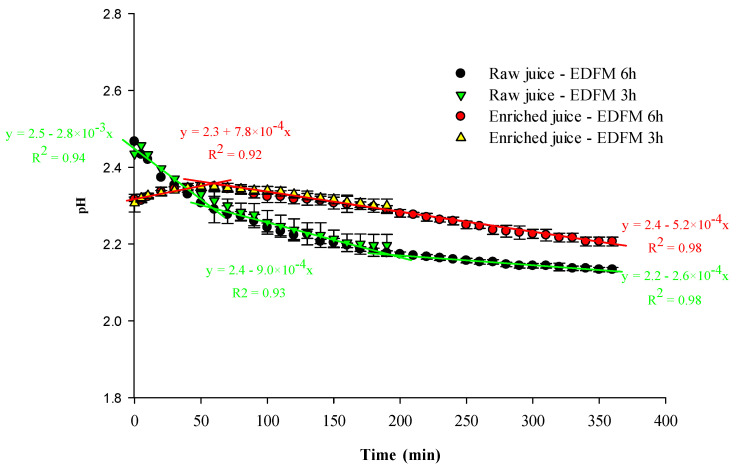
pH of the juices as a function of time during EDFM.

The evolution of the pH of both juices after 3 and 6 h of treatment is presented in Figure 6. For the raw juice, there was a significant change in pH at the beginning and after 3 h of treatment and at the beginning and after 6 h of treatment (*p* ≤ 0.001). For the enriched juice, there was no significant change in pH at the beginning and after 3 h (*p* = 0.710) of treatment, but a significant change appeared after 6 h (*p* ≤ 0.001). However, the pH evolution during the 6 h-EDFM treatment followed the same trend than for the 3 h-EDFM. The pH evolution of juices followed a distinctive tendency, as testified by the different slopes presented in Figure 6. A three-step raw juice pH evolution was observed, while a two-step enriched juice evolution was identified (Figure 6). Between 0 and 50 min, the pH of the raw juice decreased while the pH of the enriched juice was relatively stable. Then, between 50 and 180 min, the pH of both juices decreased, especially the raw juice. Finally, between 180 and 360 min, the pH of both juices continued to decrease but the enriched juice pH decreased more as attested by the different slopes (5.2 × 10^−4^ pH unit/min vs. 2.6 × 10^−4^ pH unit/min). According to the configuration, the evolution of pH during the process could indicate water splitting (WS) phenomena at both IEMs. Indeed, as already described in a previous study [32], WS is expected to happen first and mainly at the AEM explaining the higher pH decline of the raw juice during the first 50 min [9]. The pH decline of the enriched juice after 50 min could be due to the high electrical mobility of H^+^ ions (325 × 10^−5^ cm^2^/s·V) [9], the important H^+^ ion generation at the AEM, and their migration through the FM toward the enriched juice compartment. The pH drop was less important after 50 min for the raw juice and could indicate a partial compensation of the H^+^ generated at the AEM by the OH^−^ ions generated at the CEM by WS. 

Conductivity 

**Figure 7:**
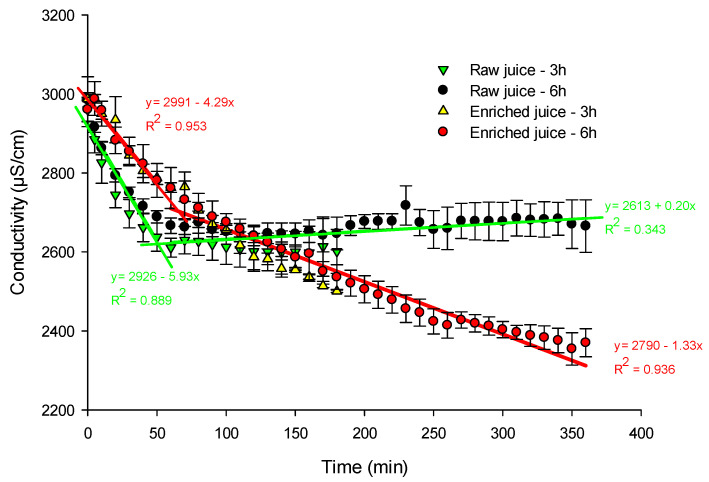
Conductivity of the juices as a function of time during EDFM.

The enriched juice conductivity decreased all along the process starting from an average conductivity of 2974 ± 19 μS/cm to a conductivity of 2501 ± 3 μS/cm (decrease of 16%) and 2370 ± 36 μS/cm (decrease of 20%), respectively, after 3 and 6 h of EDFM (Figure 7). The conductivity reached at the end of the treatment was statistically different from the conductivity at the beginning of the treatment (*p* ≤ 0.001). The raw juice conductivity decreased especially during the first 50 min starting from an average conductivity of 2973 ± 18 μS/cm to a value of 2601 ± 56 μS/cm (a decrease of 12%) after 3 h and to a conductivity of 2666 ± 67 μS/cm (a decrease of 10%) after 6 h of EDFM. For the first 50 min, the important decrease in conductivity of both juices can be explained by the depletion of most of its cations (mainly K^+^, in highest concentration in cranberry juice, see Table 1) and H^+^ ions (with stronger electrical mobility than K^+^ ions: 325 × 10^−5^ cm^2^/s·V vs. 67 × 10^−5^ cm^2^/s·V, respectively [9]) generated at the AEM by WS, which migrated toward the enriched juice and then the electrolyte. However, the conductivity drop of the enriched juice after 50 min was less important (as testified by the equation coefficient (−1.33 vs. −4.29 µS/cm·min) and could indicate that OH^−^ ions were generated at the CEM and compensated the H^+^. Meanwhile, once deprived of an important part of its ions, the raw juice conductivity stabilized by the more important H^+^ generation due to the WS at the AEM, along with some compensation by OH^−^ ions generated at the CEM. 

The pH and conductivity of both raw and enriched juices changed significantly during the 6-h EDFM treatment, with the raw juice showing a more pronounced pH decline initially and both juices experiencing a decrease in conductivity, primarily due to ion depletion and water splitting phenomena. Regardless of these phenomena, anthocyanin migration in cranberry juice was constant across the tested time intervals. 

### 3.2. Membranes Characterization

#### 3.2.1. Optical Microscopy and Membrane Cross-Section Visualization

**Figure 8:**
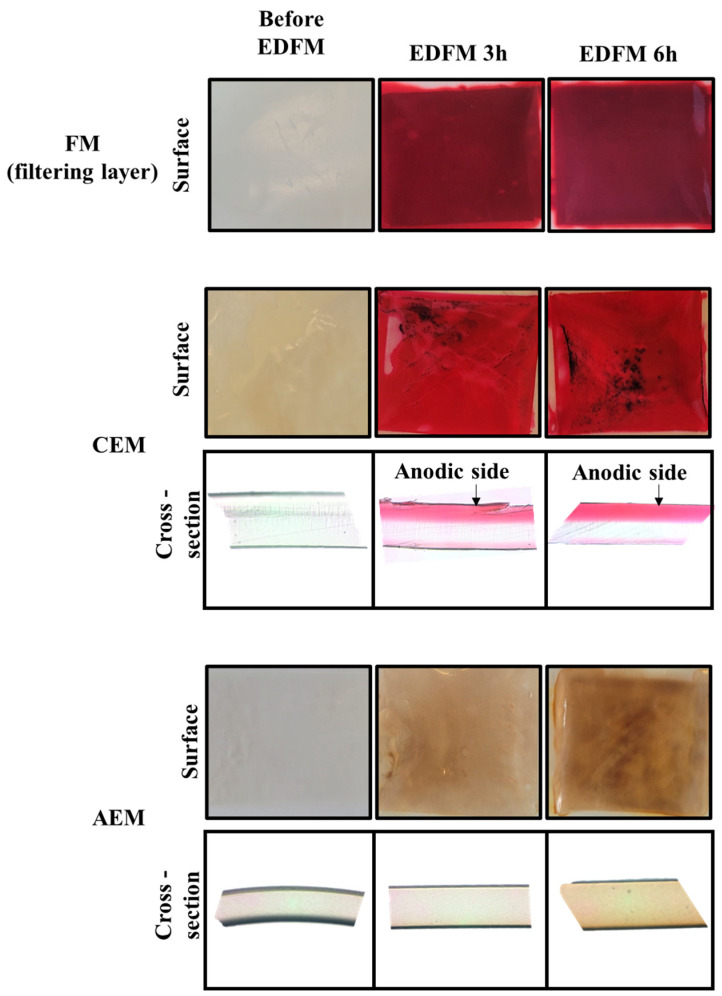
Photos of the surface of AEM, CEM, and FM and optical microscopy of cross-sections of AEM and CEM before, after 3 or 6 h of treatment.

Photos of membranes before EDFM, after 3 and 6 h of treatment, as well as optical microscopy of cross-sections of AEM and CEM before and after 3 or 6 h are presented in Figure 8. Due to a significant density of pores which creates diffraction, no information could be extracted from the cross-sections of FM, therefore they are not presented. However, FM became red, indicating the presence of anthocyanins. The color did not seem more intense after 6 h of treatment. These results seemed to indicate that the anthocyanins interacted with FM but were not fouled more after a longer treatment. On the other hand, AEM and CEM fouling have been extensively studied [9,11]. As previously observed, CEM became red indicating the presence of anthocyanins and a black deposit was visible. The color intensity did not increase between 3 and 6 h of treatment. For the CEM, on the cross-section picture, after 3 and 6 h of treatment, the red coloration was visible on the edge of the membrane, indicating a localized anthocyanins presence. However, this coloration was not visible in the center of the membrane, suggesting that interactions between the membrane and anthocyanins were mostly localized on the anodic side. For the AEM, a yellow-brow coloration appeared after 3 h of treatment and deepened after 6 h of treatment in all the membranes. Thus, a longer treatment seemed to affect the AEM more than the CEM. Also, for the AEM, the coloration was not localized at the edge of the membrane and could indicate that anthocyanins crossed the AEM and stayed inside it. Membrane desorption was conducted to ascertain the cause of the coloration (see Section 3.2.4). 

#### 3.2.2. Membrane Conductivity and Thickness

**Figure 9:**
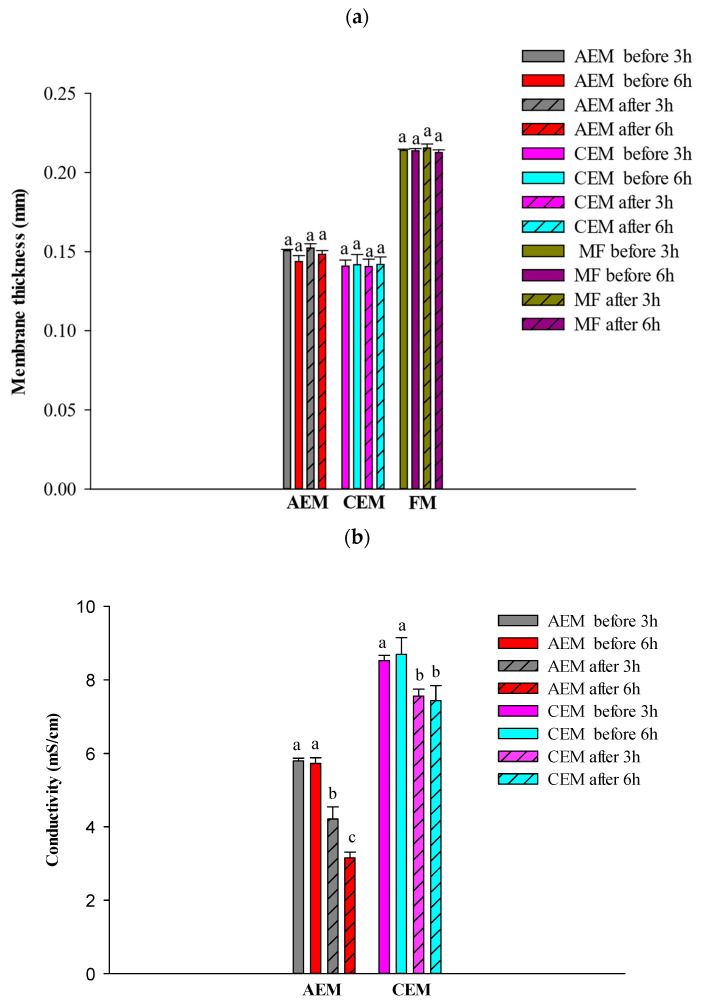
(**a**) Membranes (AEM, CEM, FM) thickness before and after 3 or 6 h of EDFM treatment and (**b**) membranes (AEM, CEM) conductivity before and after 3 or 6 h of EDFM treatment. For the same membrane, values followed with different letters (a,b,c) are significantly different (ANOVA, Tuckey) at *p* < 0.05.

No significant membrane thickness differences were observed, whatever the duration of the EDFM process (3 or 6 h) (Figure 9a). The conductivity of the AEM notably decreased after 3 h of treatment by a factor of 1.3 (*p* < 0.05), and even further after 6 h of treatment with a decrease by a factor of 1.8 (equivalent to an average conductivity loss of 45%) (Figure 9b). Phenomena like fouling, scaling, or membrane deterioration are known to decrease the conductivity of membranes and could explain the conductivity losses of the AEM. Previous studies have already described conductivity losses after contact with cranberry juice and wine (both containing polyphenols), and the conductivity of AEMs decreased by a factor of 1.6 to 4 depending on the AEM and the time of membrane contact [14,33]. This decline in conductivity was attributed to π-π (stacking) and the electrostatic interactions of anthocyanins with ion-exchange material [14]. Interestingly, the loss in conductivity for the CEM was similar after 3 or 6 h of treatment. The conductivity decline was around 15%. Conductivity diminution below 30% has been accredited in previous studies to divalent ion (from the juice) replacement with lower conductivity inside the membrane [34]. Conductivity decline by a factor of 1.1–2 for different CEMs has already been studied for juice and wine, but most of these studies were done after longer membrane time contact with the juice or wine [13,14]. The FM conductivity was not measured to avoid the loss of the foulant once in contact with the salt solution.

#### 3.2.3. SEM—EDX

**Figure 10:**
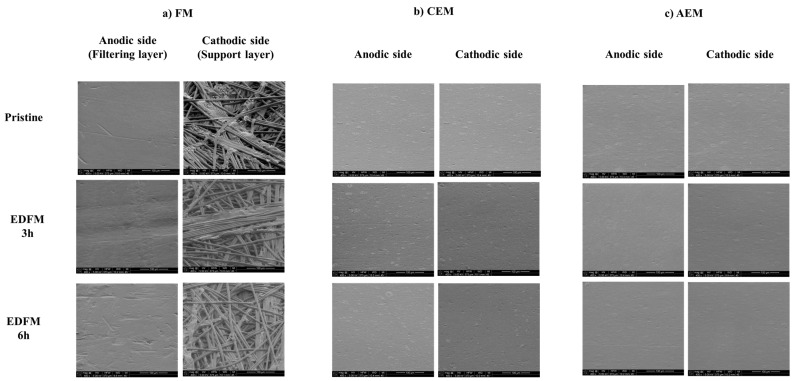
SEM images of the (**a**) FM, (**b**) CEM, (**c**) AEM anodic and cathodic sides of pristine and used membranes after 3 or 6 h of treatment.

To ascertain if scaling occurred on the membrane, SEM (Figure 10) and EDX analyses were completed on the pristine and used membranes (Table 2). SEM images did not show any sign of scaling deposits or an evolution of morphological aspects on the IEMs and FMs, as previously observed [35].

The comparison of EDX results of the pristine FM and the used FM after 3 and 6 h did not indicate the presence of mineral deposition. The elemental analysis of the FM (PVDF 250 kDa) showed the presence of carbon, oxygen, and fluor from the polymer chain of the symmetrical arrangement of hydrogen and fluorine atoms (Table 2). Concerning CEM, carbon, oxygen from the polymeric matrix, sulfur from the fixed groups of the membrane, and sodium from the counterions used to neutralize the fixed groups were identified on the membranes. The elemental analysis displayed a different mineral deposition on the surface of used membranes compared to the pristine CEM. The amount of sodium decreased slightly on both sides of the membrane compared to the pristine CEM. These results suggested that the counterions were replaced with another ion. The results were in accordance with the low decline in conductivity of the CEM due to counterions replacement (Section 3.2.2). Given the standard deviation and the small increment, the slight increase of chlore on the anodic side of the membrane compared to the pristine membrane did not seem meaningful. Concerning the AEM, carbon, oxygen, and chloride, the counter-ion used for their neutralization was detected. A difference in mineral content between the pristine and AEM used was observed on the anodic side of the membrane. An increase in oxygen levels on the anodic side (electrolyte side) may suggest membrane deterioration, as observed in a previous study [33], particularly in aged AEMs utilized for two years in the food industry. The decrease in chloride indicated the migration of this counter-ion through the membrane toward the juice’s compartments. 

#### 3.2.4. Foulant Identification

The total polyphenols concentration (TPC) desorbed from the FM, CEM, and AEM is presented in Table 3. The TPC did not increase between 3 and 6 h of treatments. Sarapulova et al. [16] showed that with increasing time of contact, a higher concentration of foulant was observed in AEM (by the expansion of the island-like formation of foulant around the ion-exchange particles), but in their study, the time of contact was over 6 h, which could explain the discrepancy. The TPC concentration desorbed from the membranes was the highest for the FM, then the CEM, and finally the AEM. A higher TPC concentration in the FM is in accordance with the deep red coloration observed of the membrane but also the nature of the membrane. Indeed, the porous structure of the FM, makes it more prone to fouling of polyphenols constituents than IEM. To complete this analysis, PAC and anthocyanin contents desorbed from the membrane were assessed (Table 4). For FM, the anthocyanins and PACs concentrations after 3 and 6 h of treatment were statistically similar, confirming TPC results. Thus, polyphenols did not accumulate on the surface of the membrane, suggesting that they were transiting through the FM. These results were in adequation with the pictures in Section 3.2.1. Interestingly, the most abundant anthocyanin present in the juice was not the one found most in the membrane. P3A, the fourth anthocyanin (in terms of concentration) found in the juice, exhibited the highest concentration desorbed from the membrane. This result suggested that P3A interacted more with the PVDF 250 kDa, whereas the C3G, which is the second most present anthocyanin in the juice, did not interact with it. Cyanidin seemed to interact less with the membrane than Peodin. PACs detection was already reported for IEM after a prolonged time of contact (72 h) with wine [16], and is coherent with the porous structure of the FM, facilitating the penetration and contact of the PACs with the FM. The main PACs identified and trapped on the membrane were monomers, 2-mers, and mostly polymers (probably formed by the linkages of monomers and 2-mers the most present in the juice), which is coherent with their high molecular weight, leading to their retention in the membrane. For CEM and AEM, anthocyanins and PACs were not detected. However, the red color of CEM indicated the presence of anthocyanins. The absence of detection was probably due to the anthocyanins and PAC concentration under the detection threshold of the technique. Nonetheless, it indicated that fouling occurring on these membranes was limited. The intensification of the yellow-brown coloration of the AEM observed after 6 h of treatment might result from membrane deterioration caused by alkali attack, as noted in previous findings by Solonchenko et al. [36].

#### 3.2.5. ATR—FTIR 

**Figure 11:**
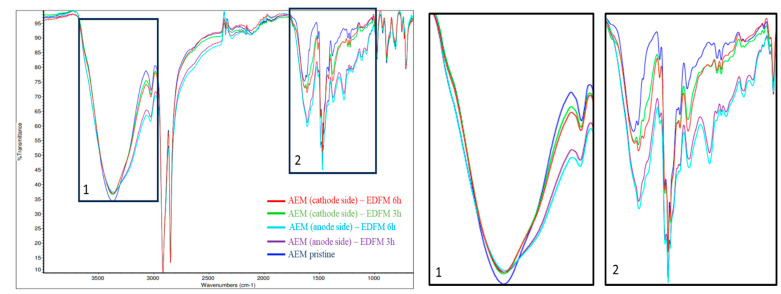
FTIR spectra of the AEM. Two regions of the spectra (1) and (2) were zoomed in to highlight the spectra changes.

The juxtaposition of FTIR spectra of pristine and used (3 and 6 h) AEM is presented in Figure 11. Two regions of the spectra (1) and (2) were zoomed in to highlight the spectra changes, which could help to identify the presence of known interactions between juice’s component and membranes (electrostatic interactions, π-π staking). The FTIR spectra of the anodic side of the AEM showed changes in the spectra, which could be caused by different types of interactions. However, the identification of cranberry juice elements can be complex and, as mentioned in Section 3.2.4. TPC desorbed from the membrane was exceedingly low. Spectra differences between pristine and used membranes could indicate structural changes in ion-exchange membrane surfaces, especially under the electric field applied in our process. Also, as mentioned by Choi et al. [32], the FTIR spectra of the used AEM membrane displayed the structural modification only on the anodic side, while no difference was observed for the cathodic side. Indeed, peak intensification and apparition in the region 1020–1220 cm^−1^ could indicate that quaternary ammonium groups of the pristine AEM membrane were transformed into tertiary amine groups under the electric field and WS [32,33]. Moreover, the band assigned to the stretching vibrations of -OH bonds was enlarged (region 3100 to 3300 cm^−1^) and could indicate the highest water content of the used AEM, as previously observed [33]. Nevertheless, for the CEM, the FTIR spectra did not show change before and after the 3 and the 6 h treatment. Therefore, the FTIR spectra did not indicate the presence of interactions between anthocyanins and the membrane and no structure change of the CEM. It is known that CEM is more stable in terms of structural change than AEM under a strong electric field [32]. 

**Figure 12:**
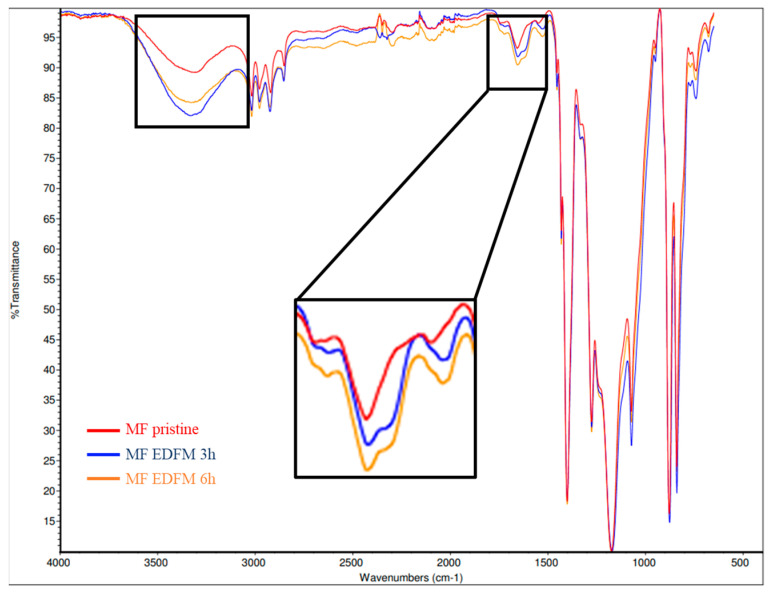
FTIR spectra of the filtering layer of the PVDF 250 kDa (FM).

For the filtering layer of the PVDF 250 kDa, an intensification of the peak at 3400 cm^−1^ and 1600 cm^−1^ after 3 h was visible compared to the pristine membrane (Figure 12: FTIR spectra of the filtering layer of the PVDF 250 kDa (FM)). The intensification did not increase after 6 h of treatment. The hydrogen bond established between the hydroxyl/carboxyl of the foulant (anthocyanin) weakens the -OH covalent bond and leads to a decrease in wavelength number by 50 cm^−1^ and an enlargement [14]. At 1600 cm^−1^, the widening and intensification of the band were also an indicator of the aromatic cycle of anthocyanins for FM. Interestingly, the support layer FTIR spectra did not show change before and after 3 and 6 h of treatment. These results confirmed that the anthocyanins were probably in transition as they migrated rather than fouled in the membrane. 

Together, these analyses provided interesting information on anthocyanin behavior at membrane interfaces and the state of the membranes. Time did not affect the fouling of the CEM, AEM, and FM (for this system and the two durations tested). But for AEMs, the results highlighted structural changes in the used membranes. In the case of CEMs, coloration indicated that a small amount of anthocyanins were trapped on the membrane surfaces. Regarding FMs, both anthocyanins and PACs were desorbed, with most foulants localized in the filtering layer rather than the support layer. 

### 3.3. Electrodialytic Parameters

#### 3.3.1. Global System Resistance 

**Figure 13:**
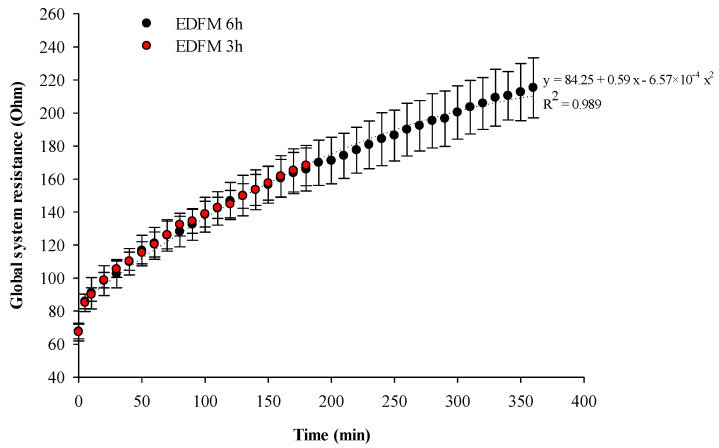
Global system resistance during 3 and 6 h of EDFM.

The global system resistance (GSR) during treatments is presented in Figure 13. The global system resistance increased from 67.71 ± 4.45 Ω to a final resistance of 215.26 ± 18.17 Ω after 6 h (corresponding to a 3.2 folds increase) and with a similar trend increased from 67.42 ± 5.41 Ω to 168.19 ± 12.25 Ω after 3 h of treatment (corresponding to a 2.5 folds increase). Elevation of resistance could be due to membrane fouling, membrane deterioration, and the WS phenomenon. Indeed, all filtration membranes and cationic membranes presented a red coloration (detailed in Section 3.2.1 as if anthocyanins had interacted with membranes. Similar fouling was detected for the same configuration and electrical field. Indeed, a previous study (Bazinet et al., 2009) [9] demonstrated a similar initial resistance (68.5 ± 5.0 Ω) but the final resistance (99.2 ± 7.0 Ω) after 4 h of treatment was lower than in the present study. 

#### 3.3.2. Relative Energy Consumption (REC)

The relative energy consumption/g of anthocyanins was 1525.6 ± 187.4 Wh/g of migrated anthocyanins in the enriched juice after 3 h and 1498.7 ± 78.9 Wh/g of migrated anthocyanins in the enriched juice after 6 h of treatment. The REC was similar after 3 or 6 h of treatment (*p* = 0.830) indicating that the EDFM needed the same energy for the migration of the same quantity of anthocyanins through time, and no fouling hampered the migration. 

Considering the REC and resistance energy, results suggest that anthocyanin migration was consistent across the tested time intervals, regardless of membrane fouling, membrane deterioration, and WS phenomenon.

## 4. Discussion

### 4.1. Impact of EDFM Duration on Juice Composition and Process Efficiency 

The anthocyanin concentrations in the juices were affected by the duration of the process. Indeed, a longer duration allowed the migration of more anthocyanins in the enriched juice compartment. Thus, two anthocyanin-impoverished juices (−18.94 ± 1.23% and −30.70 ± 2.97%) and two anthocyanin-enriched juices (26.21 ± 3.70% and 44.21 ± 5.36%) were produced. The PAC concentration remained stable before and after the treatment, regardless of the duration of the treatment with an initial concentration of 137.51 ± 3.02 mg/L and a final concentration of 142.01 ± 4.41 mg/L and 140.76 ± 2.65 mg/L after 3 and 6 h, respectively. The color of juice was also not affected by the EDFM duration as they remained stable before or after the treatment regardless of the duration of the treatment. 

Overall, the OA concentrations of the juices compartment were similar after 3 or 6 h of treatment (loss of −6.34% ± 1.45) but the concentration of malic acid declined from 3 to 6 h of treatment because it exhibited a faster migration rate through the AEM compared to citric acid and quinic acid (other organic acids initially present in the juice at high concentrations), of which the migration was hampered by steric hindrance. 

Nevertheless, the demineralization process was impacted by the treatment duration, which, in turn, affected the brix of the juices. A longer duration facilitated the migration of more minerals from the juices toward the electrolyte solutions. Indeed, in accordance with the configuration and the membranes stacked, the ions could migrate from the juices through their respective IEMs. Thus, the raw juice is quickly depleted of the most abundant cations K^+^ (88.47% after 6 h of treatment) while the enriched juice demineralization was limited by the provision of K^+^ from the enriched juice for the first 3 h (39.1% of demineralization), but the concentration finally decreased (74.2% after 6 h of treatment). 

To the best of our knowledge, for the first time, the present study demonstrated that the EDFM duration did not impact the process efficiency for anthocyanin migration, as demonstrated by the linear migration of anthocyanins toward the enriched juice compartment and the similar REC after 3 or 6 h of treatment at 30 V. However, the global resistance of the system continuously increased during the EDFM process (a 2.5-fold increase after 3 h and a 3.2-fold increase after 6 h of treatment). By examining the juice’s conductivity and pH evolution, this GSR elevation could be explained by the occurrence of WS and the subsequent AEM deterioration. Indeed, when the LCD is reached, WS occurs because the ion concentration at the surface of the IEM on the dilute side approaches zero. The WS occurring at the IEM, and the ensuing AEM deterioration, could induce serious changes in EDFM performances [37]. Overall, the global decrease in pH of both juices could be explained by the higher WS occurring first and mainly at the AEM [38] and the generation of H^+^. The conductivity decrease of both juices at the beginning of the treatment could be explained by the rapid migration on ions toward the electrolyte solution. Then, after 50 min, the stabilization of the raw juice’s conductivity occurred, and the less significant decline in the conductivity of the enriched juice and the relatively stable pH of the enriched juice may suggest that WS also occurred at the CEM, which would counterbalance the initial conductivity changes. As mentioned in Section 2.2, a voltage of 30 V (corresponding to an electric field strength of 15 V/cm) was used to reproduce the study conditions of Bazinet et al. [9] and to allow further comparisons. Similar pH and conductivity behavior were reported by the authors, and WS was also mentioned but not calculated. To confirm a possible WS phenomenon, the limiting current density (LCD) was assessed using the method of Cowan and Brown [39], and was 38.5 ± 7.5 mA·cm^−2^ with an associated voltage value of 29.9 ± 5.5 V with the whole electrodialysis cell. These results confirmed that the limiting current density was reached and water splitting at the membrane–solution interface boundary appeared. In EDFM, since the only driving force is the electric field, the higher the current density, the higher the migration of the charged molecule. The electric field strength or current density impact on EDFM efficiency was previously reported by Aider et al. [40] for the migration of chitosan, by testing three different voltages (5, 10, 20 V corresponding to three average electric field strengths of 2.5, 5, and 10 V/cm). Comparable to the linear anthocyanin migration highlighted in the present study, the authors showed a linear chitosan oligomer electromigration that was increased by increasing the electric field, and by applying a higher voltage, chitosan with a higher degree of polymerization could easily migrate. Similarly, other studies have shown a linear migration of peptides [41] with a higher migration of targeted molecules under a higher electric field. However, these studies were performed under the LCD, which could have an important effect on the global system resistance. Under the LCD, Aider et al. [40] demonstrated that by increasing the electric field strength, the resistance decreased. On the contrary, in the present study, by reaching the LCD, WS occurred at the IEM along with AEM deterioration and consequently, the GSR rose [37]. Also, reaching the LCD could have an important effect on the migration of charged molecules if the pH of the compartment changes. In the present study, anthocyanin charges were not affected by the pH decrease, remaining positively charged, thus the migration was not modified. Likewise, pH changes related to WS during the EDFM process have been observed in different studies without affecting the migration of the target molecules. Doyen et al. [42] tested the impact of water splitting on AEM during EDFM and showed an increasing peptide migration but a stagnation of the selectivity in the limiting current density regime. The EDFM cell configuration was different from the one in the present study, but in both systems the AEM was set near the anode. By measuring the electrical potential difference at the IEM interface, Doyen et al. [42] confirmed that AEM was affected by LCD, particularly at an electric field strength of 3.6 V/cm. Doyen et al. also highlighted that high electric field strengths decreased the selectivity of the process for low molecular weight (MW) peptides by allowing high MW peptides to migrate through the filtration membrane. In the present study, the anthocyanins MW and chemical structures were relatively similar, and the selectivity was not affected by the LCD. Also, this non-classic current condition was studied in an ED system with nanofiltration (NF) for the recovery of lactic acid and demineralization [30]. The author affirmed that the overlimiting current condition led to a higher lactic acid recovery rate of acid whey. Thus, for the present study, anthocyanin migration could have been helped in the LCD regime. However, the non-classic current conditions must be considered in the interpretation of fouling mechanisms. 

### 4.2. Impact of EDFM Duration on Membrane Fouling

Membrane characterization and fouling identification allowed us to assess that EDFM duration did not influence the fouling of IEM and FM. Regarding the FM, the same quantity of anthocyanins but also PACs were desorbed from the membrane after 3 and 6 h. In addition, the FTIR results indicated that most of the foulant was localized in the filtering layer, and not in the support layer. Therefore, the anthocyanins remained in transit within the FM, and their interactions with the FM did not hinder the enrichment process. The FM selected in this study have already achieved the highest anthocyanin enrichment compared to other FM in aprevious study ([10]). As for the FM, the FTIR spectra did not show change in the CEM before and after the 3 h- and 6 h-treatment. Therefore, the FTIR spectra did not indicate the presence of interactions between anthocyanins and the membrane, nor any structure change of the CEM. Nevertheless, the red coloration and the desorption of polyphenols from the membrane suggested the presence of a minor quantity of anthocyanins trapped on the membrane surfaces, regardless of the treatment duration. Therefore, the relatively low fouling neither hindered the anthocyanin enrichment nor affected product quality, as significant enrichment was achieved. However, a previous study on anthocyanins fouling ([13]) highlighted that CEM with high ion-exchange capacity, thickness, and presence of meso and macropores in their structure were more prone to fouling. The selection of CEM should take these parameters into account.

Similarly, the desorption of polyphenols from the AEM indicated the presence of a small amount of anthocyanins trapped on the membranes’ surfaces, regardless of the duration of treatment. However, results indicated changes in the AEM state during the EDFM, which were enhanced by a longer duration. The FTIR spectra changes on the AEM anodic side, the important decrease in membrane conductivity after 3 h, and even more after 6 h, highlighted the structural modification of the used membrane. In a previous study [33], an increase in oxygen on the anodic side (electrolyte side) was also observed by elemental analysis on deteriorated AEM (used for two years in the food industry), which is in adequation with FTIR results (detailed in Section 3.2.5). Sata et al. [43] explained also that the deterioration of AEMs was more intense for membranes in alkaline pH, like the conditions of our study, with the migration of the OH^−^ ions through the membrane and the diffusion boundary layer. The brown color of the AEM could also testify to anthocyanins changing in their yellow chalkon anion. Thus, results also highlighted that anthocyanin chemical forms at the interface of the membranes changed during EDFM. 

**Figure 14:**
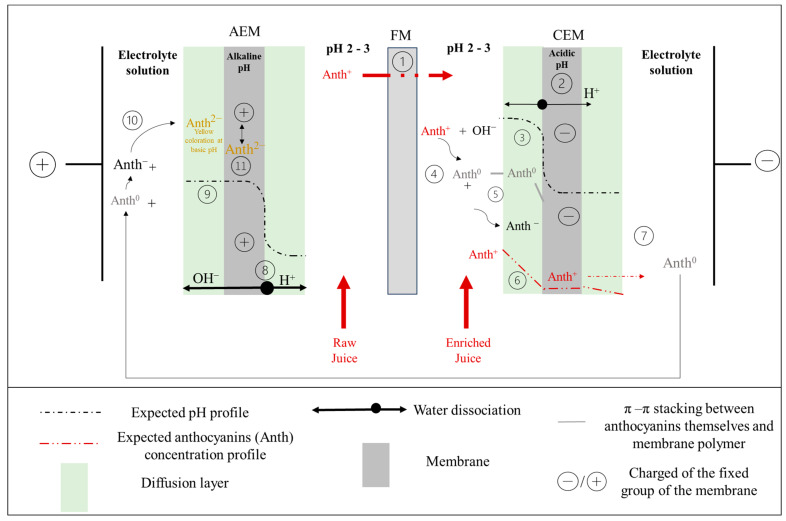
Schematic drawing of water dissociation site at the AEM and CEM, and associated pH-shift and anthocyanin charges change.

The membrane characterization, anthocyanins desorption results, and the determination of the LCD allow us to hypothesize the following anthocyanins’ behavior at the membrane interface. The anthocyanins from the juice at pH 2.5 were red flavynium cations (Anth^+^), and under the electric field crossed the FM toward the enriched juice (Figure 14 (1)). As EDFM treatment progressed, the raw juice compartment became demineralized and lost anthocyanins. Fewer and fewer ions were available to carry the current through the membranes, as testified by the conductivity decrease of the juice (Section 3.1.4). Additionally, the process was carried out above the LCD, so the condition promoted this effect: the ion concentration at the surface of the AEM on the diluate side (the raw juice) approaches zero. Associated with this effect, CEMs were fouled by the anthocyanin as testified by the red coloration (Section 3.2.1). All these conditions led to WS. Hence, anthocyanins (Anth^+^) encountered the anodic side of the CEM, where, after a certain time (50 min), a weak WS occurred (Figure 14 (2)). The generation of OH^−^ ions, at the anodic side of the membrane induced a localised pH increase of the juice surrounding the CEM (Figure 14 (3)). Hence, part of the anthocyanins reacted with the OH^−^ ions and could change in their neutral (Anth^0^) or anionic form (Anth^-^) (Figure 14 (4)), inducing the black deposit and π-π staking as the main interactions with the membrane (Figure 14 (5)). However, a small part of anthocyanins (Anth^+^) interacted like a counter-ion with the fixed group of the CEM under the electric field and could cross the CEM (Figure 14 (6)). Indeed, due to the WS, the generation of H^+^ ions, and the Donnan effect (which led to the exclusion of the OH^−^ co-ions), the pH inside of the CEM is more acidic [11]. Therefore, anthocyanins acquired positive charges (Figure 14 (6)), which facilitate their migration toward the cathode side, and in the electrolyte solution (Figure 14 (7)). Thus, at the end of the treatment, when the electric field is stopped and the WS did not occur, the deeper coloration on the anodic side of the CEM (cross-section visualization—Section 3.2.1) could be explained by the accumulation of anthocyanins due to electrostatic and π-π staking interactions. While the lighter coloration on the cationic side of the CEM could be due to the remaining anthocyanins going through the CEM in which they were blocked. Once in the electrolyte solution (at pH 4–5) anthocyanins were not charged and were in their carbinol colorless pseudobase (Anth^0^) form (Figure 14 (7)). Due to water splitting occurring since the beginning of the process at the AEM, the generation of OH^−^ (Figure 14 (8)) and the Donnan effect (which led to the exclusion of the H^+^ co-ions), the internal pH of the membrane and the interface of the electrolyte solution shifted to a higher pH (Figure 14 (9)) [11]. Consequently, as soon as anthocyanins were in contact with the anodic side of the AEM, they acquired two negative charges and were in their yellow chalkon anion form (Anth^2-^) (Figure 14 (10)). Therefore, the negative anthocyanins were fouled inside the membrane due to electrostatic interactions with the positively charged fixed groups of the AEM (Figure 14 (11)) which could also explain the intensification of the yellow-brown membrane coloration. This important pH shift inside the membrane was already reported by Rybalkina et al. [44]. 

## 5. Conclusions

To the best of our knowledge, this study assessed for the first time the influence of EDFM duration on juice composition, process efficiency, and membrane fouling during the production of enriched juice. Our findings emphasized the significant impact of processing duration on juice composition, particularly concerning anthocyanins and mineral content. Consistent energy (1512.13 ± 19.01 Wh/g of anthocyanins) was required for the migration of equivalent quantities of anthocyanins after 3 or 6 h. This study also revealed that time did not affect the fouling of the CEM, AEM, and FM (for this system and the two durations tested). Finally, the extensive characterization of membrane characteristics in terms of membrane conductivity, optical microscopy and membrane cross-section visualization, ATR-FTIR, EDX, and foulant identification gave important information on anthocyanins behaviors at the interface of membranes when LCD was reached, but also on membrane states. Concerning AEMs, results highlighted the structural change of the used membrane. For the CEM, results indicated the presence of a small amount of anthocyanins trapped at the surfaces of the membrane. As for the FM, anthocyanins but also PACs were desorbed from the membrane, but the cross-section of the membrane and FTIR results indicated that most of the foulant was localized in the filtering layer and not in the support layer. Because of the instability of AEM under a high electric field, further work should be carried out under the LCD. Another study is currently being conducted to understand the impact of the different voltages on the process. Conventional AEMs with quaternary ammonium groups are inadequate for electrodialysis processes operating at high electric fields due to their poor stability in alkaline conditions. While many studies aim to develop more stable alternative AEMs [45,46], these efforts are not specifically targeted at EDFM systems. Further research is necessary to test these alternative anion-exchange membranes in EDFM process.

The present study allowed five juices with enrichment rates of 0, −31%, −19%, 26%, and 44% to be produced. Given that anthocyanins are linked to gut microbiota modulation, which positively influences gut health [47], these juices are being utilized in an ongoing study to determine a correlation between anthocyanin levels in cranberry juice and gut microbiota modulation. The enrichment of juice with valuable biomolecules using EDFM is a promising emerging technology. This study is particularly significant as it highlights key challenges, such as optimizing voltage and selecting the appropriate IEM, that must be addressed to develop an efficient process for the food industry.

## Figures and Tables

**Table 1:** Juices’ physico-chemical properties. Values followed with different letters (a, b, c, d, e) for the same line are significantly different (ANOVA, Tukey test) at *p* < 0.05.

Properties		Juices Identification				
		Initial Juice *	EDFM 3 h Enriched Juice	EDFM 6 h Enriched Juice	EDFM 3 h Raw Juice	EDFM 6 h Raw Juice
**Colorimetry**	L	27.01 ^a^**± 0.67	26.57 ^a^**± 1.50	27.26 ^a^**± 0.59	26.82 ^a^**± 1.20	27.23 ^a^**± 0.48
	a	0.84 ^a^± 0.13	0.86 ^a^ ± 0.38	0.78 ^a^ ± 0.08	1.23 ^a^ ± 0.48	1.35 ^a^ ± 0.20
	b	−0.14 ^a^ ± 0.11	−0.19 ^a^ ± 0.21	−0.35 ^a^ ± 0.08	−0.10 ^a^ ± 0.17	−0.08 ^a^ ± 0.15
	Brix	7.63 ^a^ ± 0.08	7.47 ^ab^ ± 0.06	7.4 ^b^ ± 0.10	7.43 ^ab^ ± 0.06	7.33 ^b^ ± 0.12
**Anthocyanins (mg/L)**	Cyanidin-3-galactoside	40.99 ^a^** ± 0.25	51.88 ^b^** ± 1.11	59.34 ^c^** ± 2.29	33.2 ^d^** ± 0.66	28.3 ^e^** ± 0.66
	Cyanidin-3-glucoside	1.4 ^a^ ± 0.11	1.69 ^b^ ± 0.06	1.96 ^c^ ± 0.1	1.12 ^d^ ± 0.02	0.99 ^d^ ± 0.03
	Cyanidin-3-arabinoside	40.20 ^a^ ± 0.43	50.6 ^b^ ± 1.17	57.87 ^c^ ± 2.48	32.45 ^d^ ± 0.67	27.69 ^e^ ± 0.67
	Peonidin-3-galactoside	60.29 ^a^** ± 0.49	75.64 ^b^** ± 1.6	86.37 ^c^** ± 3.32	49.08 ^d^** ± 0.93	42.12 ^e^** ± 1.04
	Peonidin-3-glucoside	7.26 ^a^ ± 0.26	9.07 ^b^ ± 0.27	10.57 ^c^ ± 0.27	6.03 ^d^ ± 0.13	5.33 ^e^ ± 0.29
	Peonidin-3-arabinoside	30.95 ^a^** ± 0.20	38.75 ^b^** ± 0.89	44.37 ^c^** ± 1.90	25.18 ^d^** ± 0.50	21.61 ^e^** ± 0.49
**Proanthocyanidins (mg/L)**	Monomers	29.30 ^a^** ± 2.83	31.54 ^a^** ± 1.14	33.03 ^a^** ± 2.07	28.08 ^a^** ± 3.02	29.43 ^a^** ± 5.65
	2–3 mers	88.42 ^a^ ± 1.08	90.45 ^a^ ± 3.0	88.93 ^a^ ± 0.98	88.89 ^a^ ± 3.16	89.14 ^a^ ± 2.52
	4–5 mers	15.49 ^a^ ± 0.32	15.61 ^a^ ± 0.89	15.04 ^a^ ± 0.32	16.08 ^a^ ± 1.09	15.47 ^a^ ± 0.52
	6–7 mers	4.30 ^a^ ± 0.23	4.42 ^a^ ± 0.67	3.75 ^a^ ± 0.6	4.56 ^a^ ± 0.59	3.91 ^a^ ± 0.35
	>7 mers	0.00 ^a^ ± 0.00	0.00 ^a^ ± 0.00	0.00 ^a^ ± 0.00	0.00 ^a^ ± 0.00	0.00 ^a^ ± 0.00
**Organic Acids (mg/L)**	Malic Acid	8079.58 ^a^ ± 63.75	7630.04 ^b^ ± 95.92	7606.96 ^b^ ± 109.42	6882.68 ^c^ ± 44.47	6288.97 ^d^ ± 308.14
	Citric Acid	14,608.18 ^a^ ± 168.62	13,454.83 ^b^ ± 44.37	13,136.23 ^b^ ± 286.75	13,685.66 ^b^ ± 113.83	13,305.57 ^b^ ± 389.34
	Ascorbic Acid	990.72 ^a^ ± 8.25	990.56 ^a^ ± 5.03	993.82 ^a^ ± 12.33	977.35 ^a^ ± 6.21	974.87 ^a^ ± 23.34
	Succinic acid ****	95.44 ^a^ ± 14.64	76.83 ^ab^ ± 1.32	78.59 ^ab^ ± 7.33	64.26 ^b^ ± 11.35	56.72 ^b^ ± 2.39
	Quinic Acid	11,103.41 ^a^*** ± 134.41	10,802.95 ^a^*** ± 85.97	11,311.29 ^a^*** ± 728.66	11,108.10 ^a^*** ± 70.55	11,139.99 ^a^*** ± 248.24
**Minerals (mg/L)**	Ca	52.72 ^a^ ± 0.21	60.56 ^b^ ± 2.78	57.16 ^b^ ± 0.45	20.77 ^c^ ± 1.20	11.25 ^d^ ± 0.99
	Cu	0.12 ^abc^*** ± 0.00	0.16 ^a^*** ± 0.01	0.19 ^b^*** ± 0.01	0.10 ^a^*** + 0.00	0.08 ^c^*** ± 0.01
	K	681.82 ^a^ ± 2.98	414.61 ^b^ ± 20.67	195.80 ^c^ ± 20.76	174.61 ^c^ ±12.92	78.87 ^d^ ± 6.22
	Mg	36.15 ^a^ ± 0.07	47.18 ^b^ ± 3.18	50.92 ^b^ ± 2.21	14.56 ^c^ ± 0.92	7.66 ^d^ ± 0.73
	Na	11.84 ^a^ ± 0.15	9.55 ^b^ ± 0.43	6.82 ^c^ ± 0.33	3.92 ^d^ ± 0.3	1.98 ^e^ ± 0.15
	P	35.89 ^a^** ± 0.22	23.52 ^b^** ± 0.29	18.58 ^c^** ± 1.14	28.75 ^d^** ± 0.55	24.25 ^b^** ± 2.23

* The initial juice composition represents the average of each repetition of the juices within the system prior to treatment, ** the statistical test was applied on transformed data, *** test with rank transformation, **** the concentrations in succinic acid were detected but some values (before the application of the dilution factor) were lower than the minimum of the calibration line (20 ppm).

**Table 2:** EDX—FM, AEM, CEM control after 3 or 6 h of treatment. (For the same atom, values followed with different letters (a,b) are significantly different (ANOVA, Tukey) at *p* < 0.05).

Side	EDFM Duration	Membranes										
		FM			CEM					AEM		
		C (Wt%)	O (Wt%)	F (Wt%)	C (Wt%)	O (Wt%)	S (Wt%)	Na (Wt%)	Cl (Wt%)	C (Wt%)	O (Wt%)	Cl (Wt%)
**Anode side**	**Control**	49.56 ^a^ ± 0.71	2.71 ^a^ ± 0.42	47.73 ^a^ ± 1.13	75.2 ^a^ ± 0.33	12.92 ^a^ ± 0.31	6.91 ^a^ ± 0.47	4.61 ^a^ ± 0.2	0.3 ^a^ ± 0.03	89.39 ^a^ ± 1.65	3.74 ^a^ ± 0.36	6.25 ^a^ ± 0.24
	**EDFM 3 h**	50.47 ^a^ ± 0.49	2.75 ^a^ ± 0.1	46.79 ^a^ ± 0.39	75.25 ^a^ ± 0.37	13 ^a^ ± 0.5	6.52 ^a^ ± 0.23	4.02 ^b^ ± 0.13	1.43 ^b^ ± 0.47	88.13 ^a^ ± 0.55	8.10 ^b^ ± 1.14	3.74 ^b^ ± 0.65
	**EDFM 6 h**	49.71 ^a^ ± 0.008	3.23 ^a^ ± 0.05	47.7 ^a^ ± 0.04	75.07 ^a^ ± 0.72	12.85 ^a^ ± 0.18	6.47 ^a^ ± 0.18	3.94 ^b^ ± 0.09	1.66 ^b^ ± 0.77	88.01 ^a^ ± 0.52	8.81 ^b^ ±1.23	3.19 ^b^ ± 0.78
**Cathode side**	**Control**	74.71 ^a^ ± 0.14	25.29 ^a^ ± 0.14	0.00 ± 0.00	75.2 ^a^ ± 0.33	12.92 ^a^ ± 0.31	6.91 ^a^ ± 0.47	4.61 ^a^ ± 0.2	0.3 ^a^ ± 0.03	89.39 ^a^ ± 1.65	3.74 ^a^ ± 0.36	6.25 ^a^ ± 0.24
	**EDFM 3 h**	74.49 ^a^ ± 0.30	25.52 ^a^ ± 0.30	0.00 ± 0.00	75.30 ^a^ ± 0.18	13.80 ^a^ ± 0.18	6.54 ^a^ ± 0.2	4.06 ^b^ ± 0.12	0.29 ^a^ ± 0.09	89.43 ^a^ ± 0.22	4.83 ^a^ ± 0.22	5.74 ^a^ ± 0.04
	**EDFM 6 h**	73.81 ^a^ ± 0.16	26.20 ^a^ ± 0.16	0.00 ± 0.00	75.38 ^a^ ± 0.28	14.07 ^b^ ± 0.34	6.34 ^a^ ± 0.19	3.93 ^b^ ± 0.05	0.28 ^a^ ± 0.09	89.56 ^a^ ± 0.25	4.65 ^a^ ± 0.36	5.80 ^a^ ± 0.11

**Table 3:** Total polyphenol desorbed from the FM, AEM, and CEM (for the same membrane, values followed with the same letter are not significantly different (*t*-test) at *p* > 0.05).

	FM		CEM		AEM	
	3 h	6 h	3 h	6 h	3 h	6 h
Total polyphenol * **(µg/cm^3^)**	1994.25 ^a^ ± 474.69	1577.44 ^a^ ± 134.02	778.99 ^a^ ± 224.01	1118.78 ^a^ ± 186.00	101.82 ^a^ ± 65.33	65.89 ^a^ ± 11.07

* Pristine membranes were desorbed, and values obtained after desorption of the pristine membranes were subtracted from the values of the used membranes.

**Table 4:** Anthocyanins and PACs desorbed from the FM (for the same anthocyanins or PAC, values followed with the same letter are not statistically significantly different (*t*-test) at *p* > 0.05).

		FM	
		3 h	6 h
**PACs** **(µg/cm^3^)**	**Monomers**	20.20 ^a^ ± 5.35	21.55 ^a^ ± 1.19
	**2-mers**	5.95 ^a^ ± 1.72	7.23 ^a^ ± 1.15
	**Polymers**	91.00 ^a^ ± 13.59	119.29 ^a^ ± 46.25
**Anthocyanins** **(µg/cm^3^)**	P3G *	7.96 ^a^ ± 2.45	8.36 ^a^ ± 1.92
	C3G *	1.79 ^a^ ± 0.52	2.02 ^a^ ± 0.36
	P3A *	9.32 ^a^ ± 2.29	8.64 ^a^ ± 2.09
	C3A *	3.40 ^a^ ± 1.01	3.58 ^a^ ± 0.69
	C3g *	ND**	ND
	P3g *	1.04 ^a^ ± 0.17	0.96 ^a^ ± 0.32

* P3G: peonidin-3-galactoside, P3g: peonidin-3-glucoside, P3A: peonidin-3-arabinoside, C3G: cyanidin-3-galactoside, C3g: Cyanidin-3-glucoside, C3A: Cyanidin-3-arabinoside.** ND, not detected.

## Data Availability

The original contributions presented in the study are included in the article, further inquiries can be directed to the corresponding author.
